# Identification and characterization of a skin microbiome on *Caenorhabditis elegans* suggests environmental microbes confer cuticle protection

**DOI:** 10.1128/spectrum.00169-24

**Published:** 2024-07-09

**Authors:** Nadia B. Haghani, Robert H. Lampe, Buck S. Samuel, Sreekanth H. Chalasani, Molly A. Matty

**Affiliations:** 1 Molecular Neurobiology Laboratory, The Salk Institute for Biological Studies, La Jolla, California, USA; 2 University of California San Diego, La Jolla, California, USA; 3 Microbial and Environmental Genomics, J. Craig Venter Institute, La Jolla, California, USA; 4 Integrative Oceanography Division, Scripps Institution of Oceanography, University of California, San Diego, La Jolla, California, USA; 5 Alkek Center for Metagenomics and Microbiome Research, Department of Molecular Virology and Microbiology, Baylor College of Medicine, Houston, Texas, USA; 6 Biology, University of Portland, Portland, Oregon, USA; Lerner Research Institute, Cleveland, Ohio, USA

**Keywords:** *Caenorhabditis elegans*, skin microbiota, microbiome, 16S RNA

## Abstract

In the wild, *C. elegans* are emersed in environments teeming with a veritable menagerie of microorganisms. The *C. elegans* cuticular surface serves as a barrier and first point of contact with their microbial environments. In this study, we identify microbes from *C. elegans* natural habitats that associate with its cuticle, constituting a simple “skin microbiome.” We rear our animals on a modified CeMbio, mCeMbio, a consortium of ecologically relevant microbes. We first combine standard microbiological methods with an adapted micro skin-swabbing tool to describe the skin-resident bacteria on the *C. elegans* surface. Furthermore, we conduct 16S rRNA gene sequencing studies to identify relative shifts in the proportion of mCeMbio bacteria upon surface-sterilization, implying distinct skin- and gut-microbiomes. We find that some strains of bacteria, including *Enterobacter* sp. JUb101*,* are primarily found on the nematode skin, while others like *Stenotrophomonas indicatrix* JUb19 and *Ochrobactrum vermis* MYb71 are predominantly found in the animal’s gut. Finally, we show that this skin microbiome promotes host cuticle integrity in harsh environments. Together, we identify a skin microbiome for the well-studied nematode model and propose its value in conferring host fitness advantages in naturalized contexts.

**IMPORTANCE:**

The genetic model organism *C. elegans* has recently emerged as a tool for understanding host–microbiome interactions. Nearly all of these studies either focus on pathogenic or gut-resident microbes. Little is known about the existence of native, nonpathogenic skin microbes or their function. We demonstrate that members of a modified *C. elegans* model microbiome, mCeMbio, can adhere to the animal's cuticle and confer protection from noxious environments. We combine a novel micro-swab tool, the first 16S microbial sequencing data from relatively unperturbed *C. elegans*, and physiological assays to demonstrate microbially mediated protection of the skin. This work serves as a foundation to explore wild *C. elegans* skin microbiomes and use *C. elegans* as a model for skin research.

## INTRODUCTION

All animals are in contact with microbial communities, termed the microbiome. Microbiomes play a significant role in shaping host physiology, adaptation, fitness, and even behavior (1–4). Organisms and their microbial inhabitants share their first interactions at the integument of the host (5). This enveloping layer, whether it be skin, membranes, or a cuticle, plays an essential role in host–microbe relationships and may act as a barrier to promote host survival. These essential roles coupled with dynamic microbial communities provide a valuable interface to study host–microbe interactions (6). Emerging studies specifically focus on the effects of single, pathogenic bacteria, which are often localized to the gut, on animal behavior and physiology (7–10). Although single-species studies allow a thorough investigation of host–microbe interactions, individual microbial species do not fully recapitulate entire communities of microbes (11). Moreover, a majority of these studies focus on pathogen invasion mechanisms of the skin or pathogen–commensal microbial interactions (12), whereas host–commensal dynamics at the skin surface remain impactful but understudied (5, 13).

This is also the case for the soil- and fruit-dwelling nematode, *Caenorhabditis elegans*. Despite the subtle implication of surface-adherent bacteria, host–microbe interaction studies at the surface layer are often focused on pathogens (14–16). In the wild, *C. elegans* are found in rotting organic matter filled with microorganisms (17–19). Therefore, an interaction between external microbes and the worm’s integument—the cuticle—is inevitable and likely a dynamic process (20), Moreover, these nematodes rely on their cuticle health to protect against environmental stressors such as harmful toxins (21), desiccants (22), or pathogens (23, 24). The function of the *C. elegans* cuticle and its many structures have been explored using mutants in the underlying genes. Animals with defective cuticle structures display a number of phenotypes including motor abnormalities, a higher susceptibility to chemically induced cuticle degradation, and protection from skin pathogens (25–28). Several modes of fungal and single-bacterial adherence and pathogenesis have been characterized (14–16), including *Leucobacter* strains known to externally invade *C. elegans* surfaces (15, 29). However, interactions involving ecologically relevant commensals and, moreover, communities of microbes that represent wild *C. elegans* encounters remain underexplored.

Nematodes are maintained on bacterial lawns in laboratory settings. Together with their genetic tools and transparent bodies, *C. elegans* have recently gained attention in microbiome studies (30, 31). In the laboratory setting, a single bacterium *Escherichia coli* (usually a single strain, OP50) serves as the animal’s food source and external environment and minimally colonizes the *C. elegans* intestine for most of its life (32). However, *E. coli* is not a substitute for a microbiome of *C. elegans* nor is it extensively associated with the nematode in the wild. A recent meta-analysis of the *C. elegans* intestinal microbiota produced a comprehensive analysis of the host’s natural gut inhabitants (17–19). This collaborative effort prompted the creation of the *C. elegans* microbiome (CeMbio), a model microbiome consisting of the 12 most abundant gut bacteria families naturally associated with *C. elegans* (33). Individual isolates from this consortium are known to affect growth, food choice behavior, and physiology, as well neural degradation through metabolite secretion (34–38). However, these studies only consider the influence and composition of CeMbio bacteria in the gut and have not examined their potential impact at other body sites, like the cuticle.

In preparation for intestinal microbial sequencing, animals are traditionally subjected to harsh bleach washes and surface-sterilization protocols to remove cuticle-resident bacteria (39). Moreover, the first study using CeMbio intensified previously established washing steps to more effectively remove bacteria that were hypothesized to still remain on the surface, even after washing multiple times (33). The examination of nematode microbiomes in the absence of stringent surface bleach sterilization may provide worthwhile insights into the effects of a comprehensive, complex, and natural microbiome on cuticle physiology and host biology.

To investigate the persistence, existence, and composition of a cuticle-resident microbiome, we develop a novel micro-swabbing tool in combination with bacterial growth curves to investigate if animals reared on a complex microbiome harbor cuticle-resident bacteria. To supplement this finding, we enumerate the abundance of bacteria on surface-sterilized animals to demonstrate that surface-sterilization protocols remove a significant number of skin-resident or associated bacteria. We then conduct 16S rRNA gene sequencing to characterize a skin microbiome on animals reared on complex microbiomes similar to CeMbio, which we call modified CeMbio (mCeMbio, Table 1). Importantly, we find that the microbiome of non-sterilized *C. elegans* is microbially distinct from the microbiome of the *C. elegans* gut and the surrounding environment. We identify skin- and gut-dominant isolates of this microbial consortium that demonstrate distinct attachment strengths to the cuticle. Finally, we show that colonization with mCeMbio and with several mCeMbio isolates confers protection for the host against noxious stimuli.

**TABLE 1:** Bacterial strains used in this study

ID	Genus	Species	Source
JUb19	*Stenotrophomonas*	*maltophilia*	mCeMbio, CGC CeMbio
JUb44	*Chryseobacterium*	*scophthalmum*	mCeMbio, CGC CeMbio
JUb101	*Enterobacter*	*sp*.	mCeMbio
JUb134	*Sphingomonas*	*molluscorum*	mCeMbio, CGC CeMbio
MYb10	*Acinetobacter*	*guillouiae*	mCeMbio, CGC CeMbio
MYb11	*Pseudomonas*	*lurida*	mCeMbio, CGC CeMbio
MYb71	*Ochrobactrum*	*pecoris*	mCeMbio, CGC CeMbio
BIGb0170	*Sphingobacterium*	*multivorum*	mCeMbio, CGC CeMbio
BIGb0172	*Comamonas*	*piscis*	mCeMbio, CGC CeMbio
BIGb0393	*Pantoea*	*nemavictus*	mCeMbio, CGC CeMbio
MSPm1	*Pseudomonas*	*mendocina*	mCeMbio, CGC CeMbio
CEN2ent1	*Enterobacter*	*xiangfangensis*	mCeMbio, CGC CeMbio
CBX151	*Leucobacter*	*celer subsp. astrifaciens*	Known skin bacterium
OP50	*Escherichia*	*coli*	Standard food

## RESULTS

### A skin swab protocol reveals that abundant mCeMbio bacteria persist on the *C. elegans* surface

**Fig 1:**
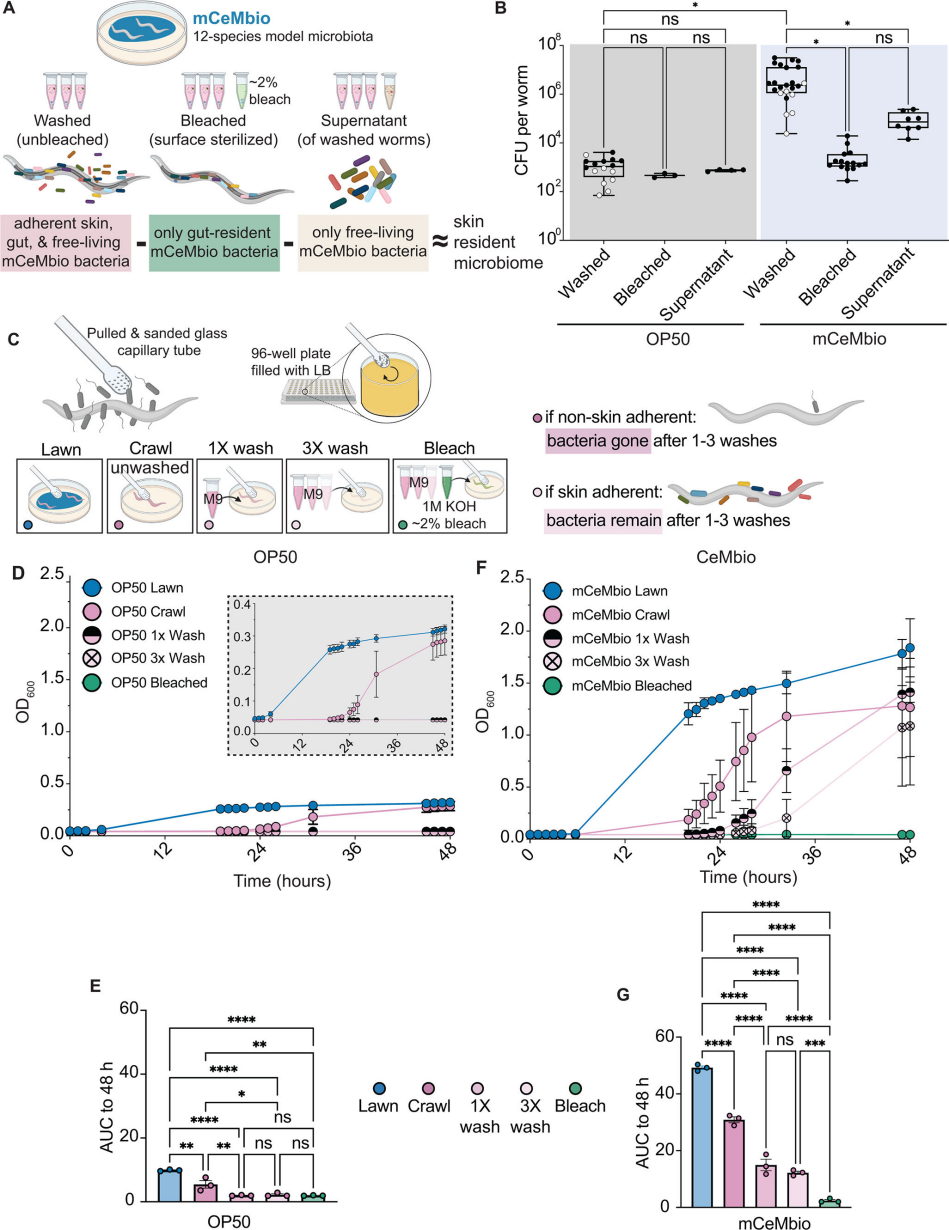
Colony Forming Unit (CFU) analysis and novel skin swab protocols reveal that abundant bacteria persist on the *C. elegans* surface (A) Diagram showing analysis of CFUs from washed animals, bleached animals, and liquid supernatants roughly estimates skin-resident bacterial abundance. (B) Average CFUs per worm in each sample type. Each dot represents an experiment with 50 worms. White dots represent experiments conducted without a bleached counterpart. Lower quartile, mean, and upper quartile marked by box and whisker plot. (C) Diagram showing skin swabbing and washing procedures used throughout Fig. 1 and Fig. 3. (D, F) The OD_600_ of swabbed bacteria from animals reared on OP50 (D) and modified CeMbio (mCeMbio) (F) is measured for 48 hours. Data shown are representative of average OD_600_ values from an individual experiment with *n* ≥ 5 animals for each condition. Mean with standard deviation in bars. (E, G) Mean area under the curve (AUC) for swabs of animals raised on OP50 (E) or mCeMbio (G) from three independent experiments, with each dot representing the average AUC for an experiment measured to 48 hours post-swab. Additional experiments are found in Fig. S1A through D. (B) Brown–Forsythe one-way ANOVA tests (for unequal SDs) with Dunnett’s T3 multiple comparisons test. (E, G): Ordinary one-way ANOVA with Tukey’s multiple comparisons test. Error bars are SEM. *P < 0.05, **P < 0.01, ***P < 0.001, and ****P < 0.0001.

We sought to directly identify the existence of a resident skin microbiome on the *C. elegans* cuticle. However, existing microbiological techniques on the nematode are designed to isolate the entirety of the animal’s microbiota rather than skin microbes. We postulated that the entire microbiota consists of gut-resident and surface-resident microbes and that these parts can be separated with varying intensities of surface-washing steps (Fig. 1A). For instance, surface-sterilization, hereafter referred to as “bleaching,” removes all skin bacteria and isolates gut-resident bacteria (Fig. 1A). Moreover, we hypothesize that animals with more skin-resident bacteria will demonstrate larger decreases in bacterial counts following bleaching. Using a colony-forming unit (CFU) assay, we assessed the existence of surface-adherent microbes on animals reared on OP50 or mCeMbio. First, we observe greater CFUs from animals reared on mCeMbio compared to OP50 (Fig. 1B). We also observe no significant difference between the number of CFUs from whole animal lysates that were serially washed or bleached after being reared on OP50 (<10^3^ bacteria per worm, Fig. 1B). However, we observe a significant decrease in the number of CFUs from whole animals after bleaching (<10^4^ bacteria per worm) compared to serially washed animals (>10^6^ bacteria per worm). From these data, we find that animals reared on mCeMbio, but not OP50, harbor skin-resident bacteria. Moreover, to account for free-living bacteria that are not in contact with the skin, we analyzed the CFUs from supernatants after a series of washes. We observe that there are OP50 and mCeMbio bacteria present in the supernatant after washing three times, which might reflect transient gut or skin microbes that have been shed, and highlight the limited extent of these methods for identifying skin-resident microbes.

Inspired by existing methods used to identify skin microbes on the surface of amphibians (40–43), we created a microscopic nematode-sized skin swab. This reusable glass tool is swabbed across the length of each animal (Fig. 1C). To eliminate non-adherent, free-living microbes and enrich for putative skin-resident microbes, we compare unwashed animals (“crawl”) to animals that have been washed once or three times. As a negative control, we include animals that have been bleached. Swabs from animals reared on OP50 indicate that OP50 grows from lawns and unwashed animals, but even washing once eliminates all skin-associated OP50 bacteria (Fig. 1D; Fig. S1A and C). Specifically, growth curves of OP50 from singly (1X) and triply (3X) washed animals are indistinguishable from the swabs from bleached animals (Fig. 1E). Swabs from animals reared on mCeMbio indicate that viable bacteria can be isolated from lawns and unwashed animals as well as animals that have been washed up to three times (Fig. 1F; Fig. S1B and D). Growth curves of mCeMbio from animals washed up to three times had ~7 fold higher levels of bacteria compared to bleached animals (Fig. 1G). These findings suggest that there are significant populations of adherent mCeMbio bacteria on the *C. elegans* cuticle that are removed by standard bleach-based surface-sterilization protocols.

### Surface sterilization alters the relative proportion of mCeMbio bacteria, suggesting distinct surface-resident communities

**Fig 2:**
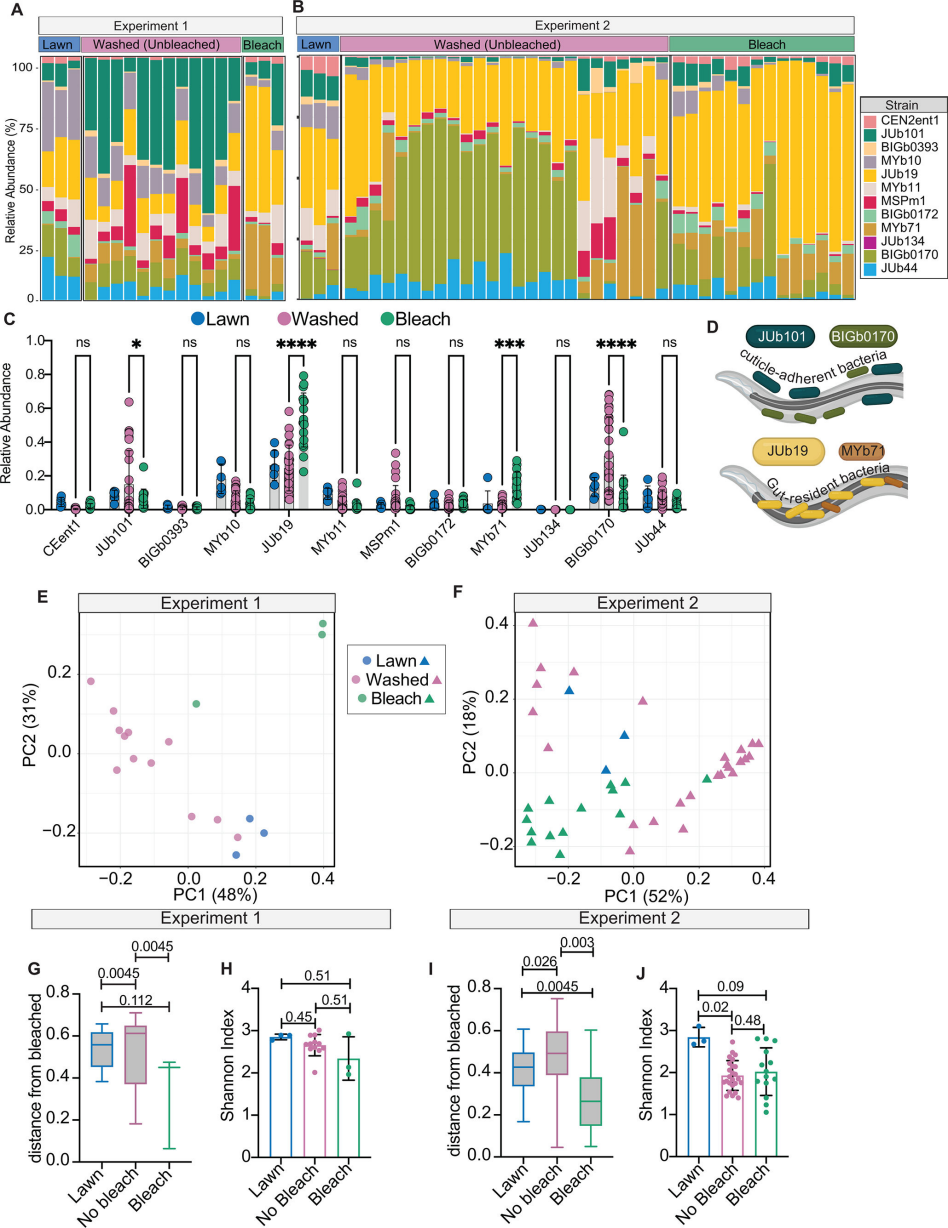
Surface sterilization alters the relative proportion of mCeMbio bacteria. (**A and B**) Proportion of reads from mCeMbio bacteria in bacterial lawns, washed (unbleached), and bleached animals from experiments 1 (**A**) and 2 (**B**). (**C**) Mean proportion of reads for lawns (blue), washed (pink), and bleached (green) N2 animals, combined from matched bleach/washed sets in experiments 1 and 2. Each dot represents one plate of animals. (**D**) Diagram of bacterial strains of interest and their expected niches, not to scale. (**E, F**) Principal coordinate analysis of Bray–Curtis dissimilarities for samples from experiment 1 (**E**) and experiment 2 (**F**). (**G, I**) Bray–Curtis dissimilarities as distance from the bleached group for experiment 1 (**G**) and experiment 2 (**I**) with pairwise PERMANOVA q-values. (**H, J**) Shannon index for each treatment in experiments 1 (**H**) and 2 (**J**) with pairwise Kruskal–Wallis q-values. (**C**) Two-way ANOVA—significant differences between bacteria and the interaction between bacteria and bleach/no bleach groups (*P* < 0.0001) with "Šídák’s multiple comparisons test" within each bacteria strain. *P < 0.05 by, **P < 0.01, ***P < 0.001, and ****P < 0.0001.

We sought to define the species of mCeMbio bacteria that reside on the *C. elegans* cuticle and determine whether they are distinct from the microbiota of the gut and the surrounding environment. We performed 16S rRNA gene sequencing on bleached *C. elegans*, serially washed *C. elegans*, and their bacterial lawns. In two separate experiments, we discovered that the bacterial communities of bleached animals have a microbiota that is distinct from those of animals that have been serially washed. In bleached animals, we observed significantly higher levels of *Stenotrophomonas indicatrix* JUb19 and *Ochrobactrum vermis* MYb71, while unbleached animals contain more *Enterobacter sp*. JUb101 and *Sphingobacterium multivorum* BIGb0170 (Fig. 2A through C). Despite significant differences between the relative abundance of bacteria across experiments 1 and 2 (Fig. S2A through C), we observe consistent differences in microbiota composition of bleached and serially washed animals, both of which are distinct from bacterial lawns (Fig. 2E and F). These data suggest that bacterial communities of bleached animals are significantly different than those of serially washed animals, as evidenced by Bray–Curtis distances (PERMANOVA, Fig. 2G and I). Furthermore, within-sample alpha diversity, expressed as the Shannon index, of each treatment group is similar, but mCeMbio bacterial lawns display greater diversity than bleached or serially washed *C. elegans* in experiment 2 (Fig. 2H and J). Our data suggest that the composition of the *C. elegans* skin microbiome is stable over time; we observe that only one bacterial species shows a significant change in abundance between 2 and 8-day-old washed (unbleached) animals, while the microbiome composition between 2 and 8-day-old bleached animals shows reduction in only two species (Fig. S2D and E). Collectively, we suggest that the microbiota of *C. elegans* with an intact skin microbiota is distinct from the microbiota of the *C. elegans* gut and further distinct from the environment on which the animals were raised. Specifically, we hypothesize that species more abundant in bleached animals (JUb19 and MYb71) are more gut-specific, whereas species more abundant in serially washed animals (JUb101 and BIGb0170) are more skin-specific.

### Specific mCeMbio microbes associate with the *C. elegans* cuticle

Informed by 16S relative abundance data, we sought to identify the effects of bleaching or serial washes on animals reared on JUb19 or JUb101. JUb101 is the only predominantly skin-resident microbe on which *C. elegans* develop at similar rates to those of OP50 and mCeMbio ((33) and our observations), and JUb19 is the gut-resident microbe with the highest relative abundance (Fig. 2C and D).

**Fig 3:**
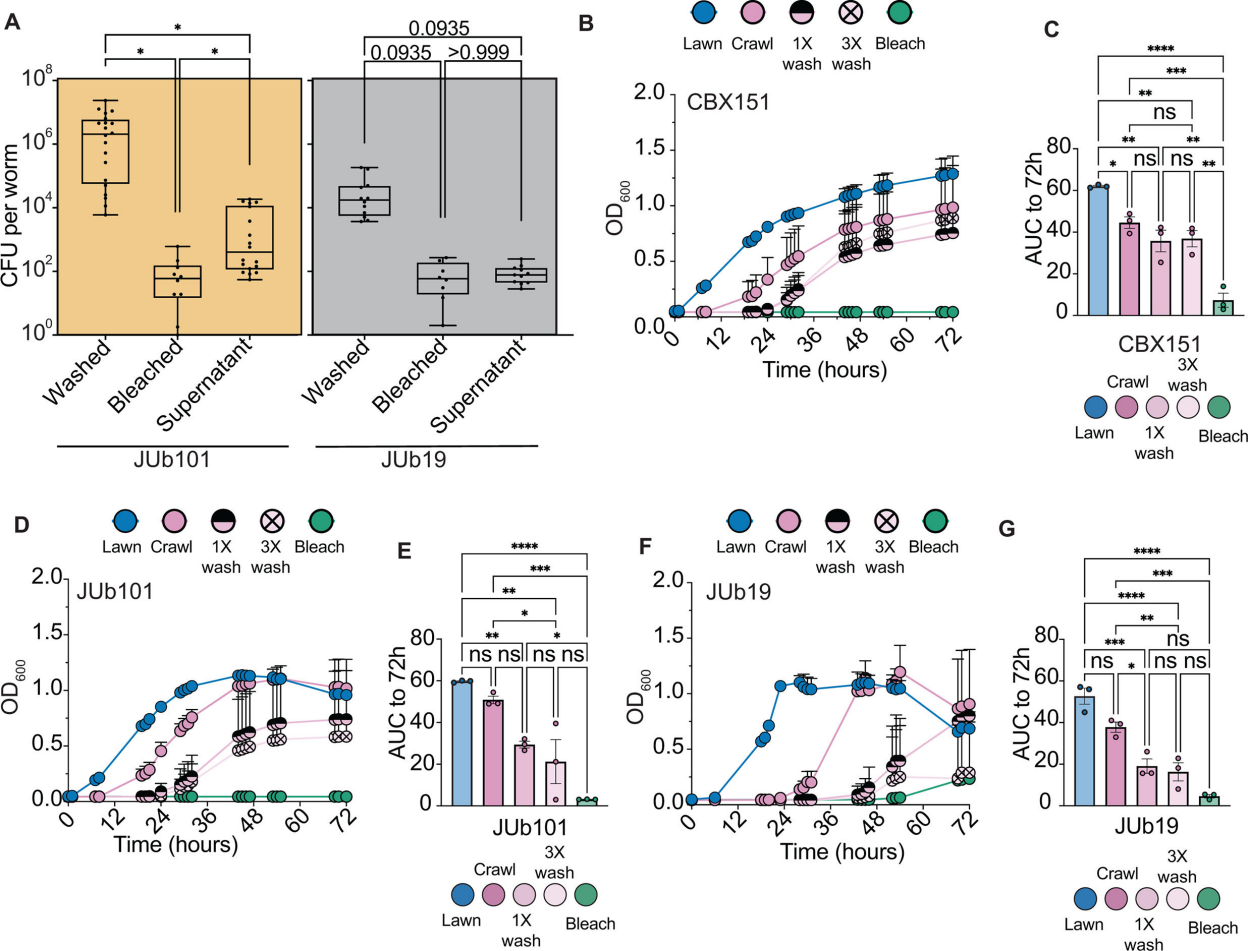
JUb19 and JUb101 display variable strengths of cuticle attachment (**A**) Average colony forming units per worm in each sample type. Each dot represents an individual plate of 50 worms, *n* ≥ 12 for *N *= 4 experiments. Lower quartile, mean, and upper quartile marked by box and whisker plot. (**B, D, F**) The OD_600_ of swabbed bacteria from animals reared on CBX151 (**B**), JUb101 (**D**), and JUb19 (**F**) is measured for 72 hours. Data shown are representative of average OD_600_ values from an individual experiment with *n* ≥ 5 animals for each condition. Mean with standard deviation bars displayed. (**C, E, G**) Mean area under the curve (AUC) for swabs of animals raised on CBX151 (**C**), JUb101 (**E**), or JUb19 (**G**) from three independent experiments, with each dot representing the average AUC for an experiment to 72 hours post-swab. Additional experiments are found in Fig. S3A through F. (**A**) Brown–Forsythe one-way ANOVA with Dunnett’s T3 multiple comparisons test. (C, E, G) Ordinary one-way ANOVA with Tukey’s multiple comparisons test. Error bars are SEM. *P < 0.05, **P < 0.05, ***P < 0.001, and ****P < 0.0001.

As in Fig. 1, we performed CFU analysis on the microbes of interest in this study. We observe more CFUs from lysates of serially washed animals reared on JUb101 (>10^6^ bacteria per worm) than lysates of serially washed animals reared on JUb19 (<10^5^ bacteria per worm) (Fig. 3A). There is no statistically significant difference between the number of CFUs from lysates of serially washed or bleached animals reared on JUb19, although JUb19 is still found on the surface. This is indicated by ~10^4^ CFU per worm found in and on serially washed animals, compared to the ~10^2^ CFU per worm found in bleached animals (Fig. 3A). Conversely, in animals raised on JUb101, there is a statistically significant decrease in the number of CFUs from whole animals after bleaching (~10^2^ bacteria per worm) compared to serially washed animals (>10^6^ bacteria per worm), indicating the presence of JUb101 as a persistent and abundant skin-resident microbe that is removed with surface-sterilization protocols, but not serial washes (Fig. 3A). To account for free-living bacteria that are loosely attached to the cuticle or transient within the worm, we analyzed the CFUs from supernatants following serial washes. We observe that JUb101 and JUb19 are both present in these supernatants, but in lower abundance when compared to washed groups.

To directly compare JUb19 and JUb101 as putative gut and skin-resident microbes, respectively, we swabbed animals reared on JUb19, JUb101, or CBX151. We include CBX151 (*Leucobacter celer* subsp. *astrifaciens*, Verde1) as a control bacterium that is known to strongly adhere to the *C. elegans* cuticle (44). We hypothesized that the patterns observed in the growth curves of swabbed skin-resident bacteria should more closely resemble those of CBX151 and mCeMbio animals (Fig. 1G), whereas the patterns observed in the growth curves of swabbed gut-resident bacteria should resemble those of OP50 (Fig. 1E). As expected, swabs from animals reared on CBX151 grow regardless of the number of washes (Fig. 3B). After 72 hours of growth, no discernable differences between swabs from animals that were unwashed (“crawl”), washed once, or washed thrice after exposure to CBX151 were observed (Fig. 3C; Fig. S3A and B). Similarly, swabs from animals reared on JUb101 grow regardless of the number of washes (Fig. 3D). After 72 hours of growth, swabs from JUb101-reared animals that have been washed once are not distinct from those of unwashed JUb101-reared animals, but are distinct from those of bleached animals (Fig. 3E; Fig. S3C and D). Conversely, swabs from animals reared on JUb19 indicate that bacterial growth from unwashed “crawl” animals is distinct from animals washed once or thrice (Fig. 3F and G). Swabs from JUb19 animals washed once or thrice are indistinguishable from swabs of bleached animals (Fig. 3G; Fig. S3E and F). Together, the patterns observed in JUb101 growth curves resemble those of CBX151, suggesting that JUb101 is also likely a cuticle-resident microbe.

### CeMbio bacteria promote wildtype and mutant *C. elegans* cuticle integrity

**Fig 4:**
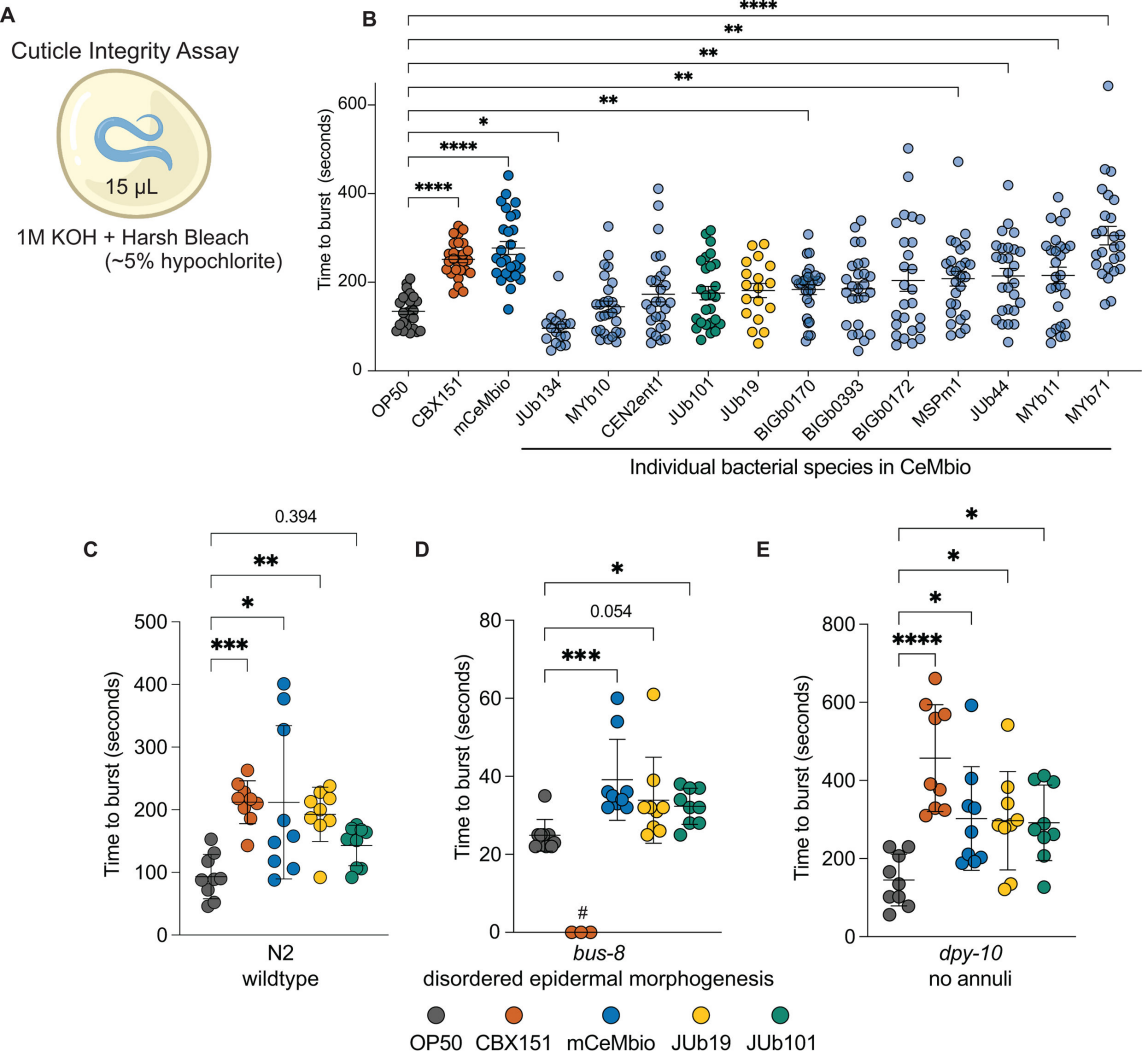
CBX151, mCeMbio, and some individual species protect wild-type and mutant *C. elegans* from harsh bleach solution (**A**) Schematic of the bleach sensitivity assay using harsh bleach solution. (**B**) The average time to burst for N2 day 1 adult worms raised on CBX151, mCeMbio, and each individual mCeMbio strain. Each dot represents an individual animal. Mean with SEM. *n* = 9 animals per experiment, *N* = 3 experiments. (**C through E**) Representative data for time to burst for N2 (**C**), *bus-8* (**D**), or *dpy-10* (**E**) animals raised on OP50, CBX151, mCeMbio, JUb19, and JUb101. Each dot represents an individual animal; *n* = 9, mean with SD. All experimental averages are given in Table 2, and normalized data from multiple experiments are given in Fig. S4B through D. (**B**) Brown–Forsythe one-way ANOVA with Dunnett’s T3 multiple comparisons test. (**C and D**) Kruskal–Wallis one-way ANOVA for unequal SDs with Dunn’s multiple comparison test (**E**) Ordinary one-way ANOVA with Dunnett’s multiple comparison test. *P < 0.05, **P < 0.01, ***P < 0.001, and ****P < 0.0001. #CBX151 was unable to be tested in *bus-8* mutants.

Given that mCeMbio contains both skin- and gut-resident bacteria, with variable strengths of attachment on the nematode cuticle, we sought to observe a potential role for skin-adherent bacteria. We hypothesized that bacteria associated with the cuticle (JUb101, BIGb0170, and CBX151 as a known skin-adherent control) would protect animals from noxious environments more than gut-associated ones (JUb19 and MYb71). To test this, we performed a cuticle resistance assay by treating animals with a harsh (5%) bleach solution (Fig. 4A). In this assay, the time to burst is a readout of cuticle integrity and a potential indication of the protective roles that bacteria may have on worm physiology (45, 46). We found that wild-type animals reared on CBX151 and mCeMbio community take significantly longer to burst compared to OP50 controls and therefore exhibit greater cuticle resistance (Fig. 4B). Animals were also reared on mCeMbio isolates individually, and we observed a range of impacts on cuticle integrity. Some bacterial species, including gut-resident *Ochrobactrum* MYb71, significantly promoted cuticle integrity with longer times to burst. Other bacteria, including the skin-associated JUb101 and gut-associated JUb19, did not alter animal burst times compared to OP50. *Sphingomonas* JUb134 was the only mCeMbio strain to significantly decrease host cuticle integrity, further supporting that most mCeMbio strains are nonpathogenic in this context (33). However, neither JUb101 nor JUb19 consistently change *C. elegans* cuticle burst time on their own (Fig. 4B). This suggests that interspecies interactions present in mCeMbio may promote cuticle protection or the N2 wildtype *C. elegans* strain lacks sensitivity in this assay and masks the subtle impact on the cuticle.

**TABLE 2:** All cuticle burst times^*a*^

	Bacteria	Set 1	Set 2	Set 3	Set 4	Set 5	Set 6
N2	OP50	93 ± 35	81 ± 28	67 ± 31	121 ± 38	265 ± 112	112 ± 30
	CBX151	212 ± 34	243 ± 67	269 ± 45	220 ± 28	NA^*b*^	NA
	mCeMbio	212 ± 122	322 ± 194	354 ± 100	346 ± 132	NA	NA
	JUb19	193 ± 43	129 ± 47	124 ± 42	81 ± 41	NA	NA
	JUb101	143 ± 32	NA	NA	NA	218 ± 49	187 ± 100
*bus-8*	OP50	25 ± 4	29 ± 3	28 ± 3	32 ± 5	30 ± 7	30 ± 6
	CBX151	Unable to test					
	mCeMbio	39 ± 10	37 ± 8	38 ± 7	50 ± 21	NA	NA
	JUb19	34 ± 11	42 ± 5	31 ± 4	39 ± 11	NA	NA
	JUb101	32 ± 5	NA	NA	NA	34 ± 11	26 ± 4
*dpy-10*	OP50	145 ± 66	151 ± 105	178 ± 117	90 ± 48	242 ± 159	193 ± 123
	CBX151	409 ± 157	481 ± 141	541 ± 86	486 ± 143	NA	NA
	mCeMbio	303 ± 132	242 ± 51	216 ± 141	396 ± 117	NA	NA
	JUb19	297 ± 126	143 ± 82	289 ± 158	296 ± 139	NA	NA
	JUb101	280 ± 102	NA	NA	NA	504 ± 193	391 ± 176

^*a*^ Average time to cuticle burst for nine animals from each genotype (N2, *bus-8,* and *dpy-10*) raised on OP50, CBX151, mCeMbio, JUb19, or JUb101. Set 1 is shown in Fig. 4C through E. All data are shown in Fig. S4 B through D.

^*b*^ NA, Not available because that group was not tested in that individual experiment.

To test this, we assessed how these bacteria affect animals with disrupted cuticle structures, thereby sensitizing the assay. We tested animals with mutations in cuticle-related genes: *bus-8, dpy-8, srf-3,* and *dpy-10* (Fig. S4A; Table S1). All genes are known to affect cuticle integrity. *Bus-8* is predicted to encode a glycosyltransferase, enabling proper cuticle molts and leading to disordered epidermal morphogenesis in strong mutants (26, 45). *Dpy-8* and *dpy-10* are collagen genes that, upon disruption, cause loss of cuticular annuli structures (27). *Srf-3* encodes a nucleotide sugar transporter, and mutants have altered cuticle surface compositions that confer biofilm resistance (28). Previous studies have demonstrated *bus-8 (e2698*) mutants as more sensitive (45) in the cuticle resistance assay, while *dpy-10* mutants have been shown to be more stiff than wild-type animals (47) and express the innate immune gene *nlp-29* at high levels constitutively (48), while also being more permeable to a Hoescht staining protocol (27), suggesting a multifaceted role for *dpy-10* in protection and permeability. Cuticle burst times of OP50-reared *bus-8* mutants are significantly shorter (time to burst = 25 seconds) than of N2 animals (time to burst = 93 seconds), as previously observed in literature (45) (Fig. 4C and D; Fig. S4A). Despite a disordered epidermal morphology and disrupted cuticle integrity in *bus-8* animals, mCeMbio consistently promotes cuticle integrity (time to burst = 39 seconds) compared to OP50 (time to burst = 25 seconds) (Fig. 4D). To normalize across multiple experiments and variable times to burst, we evaluated the fold change in burst time compared to animals raised on OP50. We observed significant differences in the fold change in burst time of *bus-8* animals raised on mCeMbio and JUb19 compared to animals raised on OP50, with mCeMbio providing 1.4-fold increases in burst time and JUb19 providing a 1.3-fold increase; JUb101 does not significantly increase the fold change in burst time but shows variability between experiments (Fig. S4B through D). Interestingly, the cuticle-adherent CBX151 caused either parental death or suppressed growth in *bus-8* animals, preventing us from testing this condition consistently in this cuticle mutant background. Cuticle burst times of OP50-reared *dpy-10* mutants are significantly longer (time to burst = 145 seconds) than of N2 animals (time to burst = 93 seconds) (Fig. 4C and E). *Dpy-10* animals raised on CBX151 (time to burst = 457 seconds), mCeMbio (time to burst = 302 seconds), JUb19 (time to burst = 219 seconds), or JUb101 (time to burst = 291 seconds) display noticeably longer cuticle burst times compared to *dpy-10* animals raised on OP50 (time to burst = 145 seconds) (Fig. 4E). This relationship is consistent with the fold change over OP50 observed for animals raised on CBX151 (3.9-fold), mCeMbio (2.3-fold), JUb19 (1.9-fold), and JUb101 (2.2-fold) (Fig. S4D). Given the consistency of increased cuticle integrity in both wild-type and cuticle mutants reared on mCeMbio compared to OP50, bacteria–bacteria interactions likely impact *C. elegans* bleach sensitivity. Moreover, *dpy-10* mutants reared on JUb101 or JUb19 promote cuticle protection compared to OP50, unmasking a subtle yet consistent impact of these bacteria on the cuticle. Despite being a single species, CBX151 is also a consistent promoter of cuticle integrity, suggesting a role for adherence to cuticle structures still present on *dpy-10* and *bus-8* mutants.

Interestingly, these cuticle structures may be critical for the adherence of some of the mCeMbio strains. We performed 16S sequencing on these sensitized strains to observe the composition of mCeMbio species present on the surfaces of the *C. elegans* mutants. We show the composition of serially washed (unbleached) *bus-8, dpy-10*, *dpy-8*, and *srf-3* mutants compared to wild-type controls collected alongside them. We observe that *bus-8, dpy-10*, *dpy-8*, and *srf-3* mutants all display reduced abundance of JUb101, the candidate skin-adherent bacterial species in this study (Fig. S4E and F). Moreover, *bus-8, dpy-8*, and *srf-3* mutants have increased abundance of JUb19, the putative gut-resident microbe in this study (Fig. S4E and F). These results allow us to begin to associate some cuticle structures with bacterial adherence.

## DISCUSSION

Bacteria–host interactions are complex and known to impact many aspects of host biology. Consequently, the study of ecologically relevant bacterial communities is often traded for the simplicity of single species. In this study, we demonstrate the importance of ecologically relevant communities of bacteria and uncover a *C. elegans* skin-resident microbiome. We reared animals on a natural *C. elegans* microbiota, mCeMbio, and discovered that most of the mCeMbio bacteria can be removed by bleaching protocols, suggesting that these bacteria may reside on the cuticle surface (Fig. 1A and B). We observed that our surface-sterilized worm CFU counts match those in previous findings (33), whereas serially washed, unbleached worms have significantly more bacteria (Fig. 1B and 3A). We conjectured that there are abundant skin-resident bacteria, approximating counts from simplified arithmetic of [# unbleached CFU] – [# bleached CFU] – [# supernatant CFU] (Fig. 1A). To supplement this finding, we developed and implemented a novel micro-swab to isolate skin microbes from *C. elegans* cuticles. We measured the growth of swabbed OP50 and mCeMbio bacteria from *C. elegans* cuticles and observed distinct and repeatable patterns of relative growth between washing conditions (Fig. 1D through G); more washes are required to remove mCeMbio bacteria from the cuticle, implying that mCeMbio contains surface-adherent bacteria.

We then conducted 16S rRNA gene sequencing to define strain-level microbial differences among serially washed and bleached animals. From these data emerged candidate gut- (*Stenotrophomonas* JUb19 and *Ochrobactrum* MYb71) and skin-dominant bacteria (*Enterobacter* JUb101 and *Sphingobacterium* BIGb0170). Although our two independent sequencing experiments revealed different proportions of microbes (Fig. S2A and C), both experiments suggest that there are distinct communities in animals with intact microbiomes compared to those without surface microbes (Fig. 2C through J; Fig. S2B). Importantly, the microbiomes from animals with cuticle-adherent microbes are distinct from those of their bacterial lawns (Fig. 2G and I), indicating some level of selection on the surface of the cuticle. Although we cannot confirm whether the composition of the supernatant from washed animals, which is composed of loosely attached bacteria, is similar to or distinct from the environment or animal surface, we focused on the more strongly attached bacteria. We further confirmed the persistence of skin-resident microbes compared to more gut-resident microbes by measuring bacterial growth from skin swabs of animals reared on these species (Fig. 3D through G). We hypothesize that multiple species within mCeMbio may also associate with the *C. elegans* cuticle to varying degrees.

None of the mCeMbio bacteria are known to be pathogenic, although the individual isolates demonstrate varying effects on host developmental rates (33). Given the symbiotic nature of these bacteria and our parallel lines of validation of surface-adherent bacteria, we hypothesized that the mCeMbio community serves a role in cuticle protection against environmental stressors. We conducted a well-established cuticle fragility assay and observed the consistent role of mCeMbio and CBX151, a known cuticle-adherent bacterium, to promote cuticle integrity in the host, which varies across the individual bacterial species in mCeMbio (Fig. 4A and B). From these data, we demonstrate the importance of studying community effects and the effects of single bacterial species on cuticle integrity. Put together, we uncover a protective effect for animals reared on a consortium of ecologically relevant bacteria.

The data from our study do not differentiate between the combinatorial effects of the mCeMbio bacteria on the skin and in the gut, and the mechanism of this protection requires further examination. Several species of bacteria found in association with *C. elegans* are known to, directly and indirectly, protect against pathogens in the worm intestine (49). Other protective benefits may be derived from diverse nutritional or metabolic mechanisms unique to the mCeMbio bacteria (33). This may provide an indirect mechanism by which gut-dominant bacteria, like MYb71, are able to protect the cuticle. Moreover, both known skin-resident bacteria and members of the mCeMbio consortium have been shown to prime the cuticle against other infections (50). This protection may even be attributed to the potential hydrogen peroxide-degrading capabilities of the mCeMbio consortium, which have been observed in strains of *E. coli* (51). Importantly, we sought to examine skin-enriched microbes and the mCeMbio consortium as a whole, which involves both bacteria–bacteria and host–bacteria interactions. This is highlighted by greater cuticle resistance in animals raised on the mCeMbio community (time to burst = 277 seconds) compared to all but one of the individual mCeMbio members, the gut-enriched strain *Ochrobactrum* MYb71 (Fig. 4B). This suggests that most single host–bacteria interactions may not fully recapitulate the extent of interactions in our system and demonstrates the importance of studying communities of ecologically relevant microbes.

In an effort to develop *C. elegans* as a model for understanding skin and associated diseases, we must make use of sensitized and protective strains. To do this, we used *C. elegans* mutants with varying levels of disordered cuticle structure and function. We found that mCeMbio, JUb19, JUb101, as well as the known skin-adherent microbe CBX151 increase protection in nearly all sensitized and protective mutants (Fig. 4C through E; Fig. S4A through D; Table 2). However, we have not confirmed that these mutant animals can be vessels for bacterial growth on their skin, given their disrupted state. We see effects on these sensitized mutants and correlate those effects to the presence of skin microbes. It is also plausible that, similar to our findings in wild-type animals, these effects could be due to nutritional supplementation from mCeMbio bacteria or otherwise mediated through the gut.

Interestingly, the washed (unbleached) *C. elegans* with mutations in cuticle genes display 16S profiles different from those of the washed (unbleached) wild-type animals. These differences are most starkly observed in the decreased abundance of putative skin-adherent microbe, JUb101, and increased presence of JUb19 across nearly all of the sensitized cuticle mutant strains. We do not have data for bleached cuticle mutant strains; due to their sensitized nature, the standard bleaching protocol was too harsh for most of the animals. Therefore, we are unable to compare between washed and bleached conditions. However, we observe that mCeMbio is able to rescue the cuticle mutants, which suggests that even small populations of the strains are protective or that the protection comes from aforementioned indirect influences.

Given the symbiotic nature of the mCeMbio bacteria, which includes 11/12 published CeMbio strains; unique cuticle structures of the nematode skin such as the annuli, alae, or invagination sites; and community effects on promoting barrier function, our data support the existence of and role for a previously unappreciated *C. elegans* skin microbiome. Our work supports the continued efforts to make the study of *C. elegans* more ecologically relevant and suggests that we continue to understand and increase environmental enrichment to improve the relevance of this model system.

## MATERIALS AND METHODS

### *C. elegans* strains and maintenance

Strains were ordered from the Caenorhabditis Genetics Center and include the following: wild-type N2; CB130 *dpy-8 (e130*); VC2985 *dpy-10 (gk3075*) II; CB6627 *srf-3 (e2689*) IV; CB6177 *bus-8 (e2883*) X. All animals were grown at 20°C under standard conditions to adulthood (52). NGM plates were seeded with either *E. coli* OP50 or mCeMbio strains, unless noted of a specific bacterium. For all experiments, L4 animals are placed on lawns and removed ~12 hours later, leaving only offspring behind. Experiments are then performed on the offspring, unless otherwise noted.

### Culture and maintenance of mCeMbio bacteria

Individual isolates of “CeMBio” bacteria, referred to as mCeMbio (gift from H. Schulenberg, available now through CGC as CeMbio, see note below in Identification of mCeMbio) and CBX151 (a gift from Marie-Anne Félix), were streaked onto LB agar plates, and individual colonies were picked and grown in LB growth media. Individual bacteria (12 mCeMbio strains, CBX151, and OP50) were allowed to grow for 2–4 days, with shaking at 4 x g at 20°C. Bacteria were maintained and prepared to OD_600_ = 0.2 by dilution in additional LB or centrifuging at 1,800 x g for 5 minutes to remove excess LB to increase the concentration of slow-growing isolates. To make mCeMbio, each of the 12 bacteria were combined at equal ratios. Two hundred microliters of the bacteria was pipetted on 6-cm NGM plates and evenly spread across the agar plate using a sterile L spreader (VWR 490007–358). Bacterial plates were left to dry in a laminar flow hood for 2 days. If not used immediately, seeded plates were stored at 4°C. We maintain glycerol stocks of each bacterium at −80°C, and these were used for each new batch of each mCeMbio strain, OP50, or CBX151 bacteria.

### Identification of mCeMbio strain JUb101

The *Lelliottia amnigena,* JUb66, genome sequenced in Houston by Buck Samuel’s group and the JUb66 genome sequenced in Kiel by Heinrich Schulenburg’s group are different. The bacterial sample deposited at the CGC as part of the CeMbio consortium is the JUb66 from the Samuel Lab (JUb66 Houston), and the one from the Schulenburg lab is now known as JUb101. We acquired our strains from the Schulenburg lab. These two genomes have identical 16S rRNA representative sequences and the same number of 16S repeats. A whole-genome comparison of JUb66 Houston and JUb101 (formerly known as JUb66 Kiel) shows only 84% similarity. To determine the identity of our CeMbio strain, formerly known as JUb66 Kiel, we used the following PCR primers and standard NEB Taq protocols.

JUb66-2270F TTGCTTCGGGTGGTGGTATC JUb66-2827R CCATGGAACTCACCCCTGTC

JUb101-1377F GAGGATGGGTATGCAGACCG JUb101-2375R GGGATCGCAATCTGACGGAT

Therefore, we call our mixture mCeMbio, a modified CeMbio consortium.

### *C. elegans* and bacterial prep for DNA extraction

Animals were reared on mCeMbio bacterial lawns for 48–82 hours after hatching. Bacterial plates (*n* = 3 for each group) were flooded with M9 and 30 animals were picked off with a glass pipette and placed into an Eppendorf tube. For unbleached animals, tubes were washed with M9 +T [M9 +25 mM tetramisole hydrochloride (Sigma-Aldrich L9756)] five times, allowing 4 minutes for animals to sink to the bottom of the tubes between each wash. For bleached animals, tubes were washed with M9 +T two times and then washed with surface-sterilizing bleaching solution (M9 +T + 2.5% hypochlorite +1M KOH) for 4 minutes. These tubes were rinsed with M9 +T twice for 4 minutes each. After washes, animals were allowed to sink to the bottom of the tube. All but 100 µL of media was removed in the final wash, and the tubes were frozen on dry ice and stored at −80°C. For “lawn” plates, 1 mL of M9 was added to the plate, and the bacterial lawns were scraped with a sterile L spreader (VWR 490007–358). Bacterial slurry was pipetted and centrifuged at 13,000 x g for 4 minutes. All but 100 µL was removed, and the pellet was frozen on dry ice and stored at −80°C. DNA was extracted following the standard NucleoSpin Tissue Kit (Macherey-Nagel 740952) protocol for animal tissue with 1 hour of incubation for the first lysis step. DNA was stored at −20°C.

### 16S rRNA amplicon sequencing and analysis

For each experiment, an amplicon library targeting the V4–V5 hypervariable region of the 16S gene was generated via one-step PCR using the Azura TruFi DNA Polymerase PCR kit, and the 515 F (GTGYCAGCMGCCGCGGTAA) and 926R (CCGYCAATTYMTTTRAGTTT) primer set was used (53). Each reaction was performed with an initial denaturing step at 95°C for 1 minute, followed by 30 cycles of 95°C for 15 seconds, 56°C for 15 seconds, and 72°C for 30 seconds. Two and a half microliters of each PCR reaction was run on a 1.8% agarose gel to confirm amplification. PCR products were purified using Beckman Coulter AMPure XP following the standard 1 x PCR clean-up protocol. PCR quantification was performed in duplicate using the Invitrogen Quant-iT PicoGreen dsDNA Assay kit. Samples were then pooled in equal proportions (20 ng DNA per sample), followed by another AMPure XP PCR purification. The final libraries were evaluated on an Agilent 2200 TapeStation (D1000 ScreenTape), quantified with the Qubit HS dsDNA kit, and each library was sequenced at the University of California, San Diego IGM Genomics Center on a single Illumina MiSeq lane with a 25% PhiX spike-in.

Amplicons were analyzed with QIIME2 v2019.10 (54). Briefly, demultiplexed paired-end reads were trimmed to remove adapter and primer sequences with cutadapt (55). Trimmed reads were then denoised with DADA2 to produce amplicon sequence variants (ASVs) (56). Taxonomic annotation of ASVs was conducted with the q2-feature-classifier classify-sklearn naïve-bayes classifier (57, 58), both the genome-derived reference 16S sequences of CeMbio strains (33) and the SILVA database (v138) (59). ASVs were then aggregated by the bacterial strain, and reads were normalized by the 16S gene copy numbers estimated by their genome assemblies (33). Bray–Curtis dissimilarities, Shannon indices, and tests of statistical significance were then calculated with the QIIME2 diversity plugin on the normalized relative abundance count data (60, 61). Raw sequence data are deposited in the Sequence Read Archive (SRA) under the BioProject accession PRJNA979901. Data are sorted into experiment 1 and experiment 2 “combine,” in which all wild-type, hermaphrodite samples, regardless of date of extraction or age, were included if they met the category “bleached” or “unbleached” (serially washed). These data are displayed in Fig. 2 and sorted by experiment. Fig. S2 displays data from combining experiment 1 and experiment 2, but only the samples in which the bleached and unbleached (serially washed) samples were prepared as a matched set on the same day and at day 1 of adulthood.

### Skin-swabbing with the 96-well plate assay

To prepare the micro-swab, a glass capillary tube (World Precision Instrument Thin Wall Glass Capillaries-TW100-4 or similar) was pulled apart over a small flame into two pieces. Heat was used to create a small closed bulb at the pulled end of each piece, ensuring there were no crevices in which bacteria or liquids may accumulate, and then the glass was allowed to cool. Then, using 600-grain sandpaper, the bulb was lightly scored to make a textured surface (~10 small circles on each side and tip). To sterilize, the swab is dipped in 70% ethanol and air-dried.

To swab a lawn, the micro-swab is dipped into the lawn on which the animals were reared and then immediately swirled in the appropriate LB-containing wells. To swab an animal with the condition “crawl,” a single animal is placed on an empty NGM agar plate using an eyelash pick and allowed to crawl for 30 seconds. Using a dissecting microscope, the animal was gently swabbed using the bulbous end from head to tail 10 times. The bulb was then dipped and swirled around in 10 circles into 200 µL of LB in a 96-well plate (CoStar 96 Flat Black). Animals that were washed once “1 x wash” were washed in M9 +T and allowed to sink to the bottom of a tube. This was repeated two more times for “3 x wash.” For animals in the “bleach” group, they were first washed with M9 +T three times, then once with surface-sterilizing bleaching solution (M9 +T + 2.5% hypochlorite +1M KOH), and once more with M9 +T. Once washed or bleached, the animals were then pipetted onto a sterile NGM plate in a small volume and allowed to crawl out of the drop before being swabbed. Once all animals were swabbed, the OD_600_ was measured using a Molecular Devices SpectraMaxPlus with absorbance = 600 nm, manually taking measurements during standard work hours. To generate the curves in Fig. 1D, F and 3B, D and F ,Fig. S1A through D and Fig. S3A through F, the individual replicate OD_600_ values at each timepoint were averaged in each condition within an experiment. Our methods make use of area under the curve analyses, which can be used to compare and quantify data from multiple growth curves (62, 63).

### Colony-Forming Unit assay

Animals were reared to day 1 of adulthood on chosen bacteria. Fifty adult worms were transferred to a 2-mL tube containing 100 µL M9 and 10–20 1 mm zirconium beads (Beadbug Z763780). Each tube of worms was washed three times for 3 minutes each with 750 µL M9 +tetramisole (25 mM), removing all but 100 µL liquid with a new pipette tip each time, allowing animals to sink to the bottom of the tube by gravity. We separated half of the tubes to be “bleached” groups, which are subjected to a 4-minute wash with surface-sterilizing bleaching solution (M9 +T + 2.5% hypochlorite +1M KOH). Bleached animals are washed for 3 minutes x 2 using M9-T to remove the bleach. On the final wash, 100 µL of the supernatant from each bleached tube is removed. Unbleached samples are washed for 3 minutes x 2 using M9-T, and 100 µL of the supernatant is removed for each unbleached tube. Each sample is homogenized using a Fisher Mixer Mill at Level 4 for 30 seconds for four cycles, totaling 2 minutes of bead beating. The homogenate was then serially diluted (1 in 100, 1000, 10000, and 100000 for each tube of 50 worms) in M9. One hundred microliters of each dilution was plated and spread with a sterile L spreader on LB agar plates. The previously removed supernatants were also diluted in M9 and plated. Plates were dried and left to grow for 48 hours. Finally, bacterial colonies were counted to quantify the original bacterial colony counts in each sample (unbleached, bleached, and supernatant of both bleached and unbleached samples).

### Cuticle fragility assay

Fifteen microliters of strong bleach solution (1M KOH and ~5% hypochlorite) is pipetted onto a single day 1 adult animal after it has crawled on a clean NGM plate for ~10 seconds. Under a dissecting microscope, the time to burst open (cuticle loses integrity) is recorded.

### Standard statistical analyses

Fig. 1, 3, 4; Fig. S1 to S4 and their statistical analyses were completed in GraphPad Prism. Ungrouped two-sample *t*-tests were used when determining the statistical significance during comparisons of two groups. With three groups and more, an ANOVA test was performed for determining significance values. When appropriate, corrections were performed for multiple comparisons or unequal variances. Any *P* value smaller than 0.05 was considered significant. The statistical tests used in Fig. 2 are described under “16S rRNA Amplicon Sequencing and Analysis.”

## Data Availability

Raw sequencing data for the 16S sequences are deposited at the National Center for Biotechnology Information Sequence Read Archive under the BioProject Accession PRJNA979901. All other data, including raw microscopy images, are available upon request.

## SUPPLEMENTAL MATERIAL

Supplemental material spectrum.00169-24-s0001.pdf Fig. S1 to S4; Table S1.

ASM does not own the copyrights to Supplemental Material that may be linked to, or accessed through, an article. The authors have granted ASM a non-exclusive, world-wide license to publish the Supplemental Material files. Please contact the corresponding author directly for reuse.
